# Walnut Oil Reduces Aβ Levels and Increases Neurite Length in a Cellular Model of Early Alzheimer Disease

**DOI:** 10.3390/nu14091694

**Published:** 2022-04-19

**Authors:** Carsten Esselun, Fabian Dieter, Nadine Sus, Jan Frank, Gunter P. Eckert

**Affiliations:** 1 Biomedical Research Center, Institute of Nutritional Sciences, Justus-Liebig-University of Giessen, Schubert-Street 81, D-35392 Giessen, Germany; carsten.esselun@ernaehrung.uni-giessen.de (C.E.); fabian.dieter@ernaehrung.uni-giessen.de (F.D.); 2 Department of Food Biofunctionality, Institute of Nutritional Sciences, University of Hohenheim, Ökozentrum, Garbenstr. 28, D-70599 Stuttgart, Germany; nadine.sus@nutres.de (N.S.); jan.frank@nutres.de (J.F.)

**Keywords:** walnut, poly-unsaturated fatty acids, PUFA, vitamin E, mitochondria, neurodegeneration, aging

## Abstract

(1) Background: Mitochondria are the cells’ main source of energy. Mitochondrial dysfunction represents a key hallmark of aging and is linked to the development of Alzheimer’s disease (AD). Maintaining mitochondrial function might contribute to healthy aging and the prevention of AD. The Mediterranean diet, including walnuts, seems to prevent age-related neurodegeneration. Walnuts are a rich source of α-linolenic acid (ALA), an essential n3-fatty acid and the precursor for n3-long-chain polyunsaturated fatty acids (n3-PUFA), which might potentially improve mitochondrial function. (2) Methods: We tested whether a lipophilic walnut extract (WE) affects mitochondrial function and other parameters in human SH-SY5Y cells transfected with the neuronal amyloid precursor protein (APP695). Walnut lipids were extracted using a Soxhlet Extraction System and analyzed using GC/MS and HPLC/FD. Adenosine triphosphate (ATP) concentrations were quantified under basal conditions in cell culture, as well as after rotenone-induced stress. Neurite outgrowth was investigated, as well as membrane integrity, cellular reactive oxygen species, cellular peroxidase activity, and citrate synthase activity. Beta-amyloid (Aβ) was quantified using homogenous time-resolved fluorescence. (3) Results: The main constituents of WE are linoleic acid, oleic acid, α-linolenic acid, and γ- and δ-tocopherol. Basal ATP levels following rotenone treatment, as well as citrate synthase activity, were increased after WE treatment. WE significantly increased cellular reactive oxygen species but lowered peroxidase activity. Membrane integrity was not affected. Furthermore, WE treatment reduced Aβ_1–40_ and stimulated neurite growth. (4) Conclusions: WE might increase ATP production after induction of mitochondrial biogenesis. Decreased Aβ_1–40_ formation and enhanced ATP levels might enhance neurite growth, making WE a potential agent to enhance neuronal function and to prevent the development of AD. In this sense, WE could be a promising agent for the prevention of AD.

## 1. Introduction

Walnuts are an important component of the Mediterranean Diet, which has been shown to prevent Alzheimer’s disease (AD) [1,2]. Clinical studies revealed that the consumption of walnuts increases cognitive function in the elderly [3,4,5,6]. However, the more recent Walnuts and Healthy Aging (WAHA) study showed that supplementation of walnuts for 2 years had no effect on the cognition of healthy elders. On the other hand, brain fMRI and post hoc analyses suggested that walnuts might delay cognitive decline in subgroups at higher risk [7]. Recently, we have reported that a walnut diet in combination with encouraged physical activity improves cognition and affects the oxylipin profile in the brain and liver of aged NMRI mice [8]. However, mitochondrial dysfunction, which represents a common final hallmark of both brain aging and neurodegeneration [9], was not altered in aged mice fed with walnuts [8]. SH-SY5Y-APP695 cells were transfected with the human amyloid precursor protein (APP)-coding region APP695, effectively altering the procession of APP and leading to moderately increased production of Aβ [10,11]. SH-SY5Y cells are among the few cell lines that can convert α-linolenic acid (ALA) to eicosapentaenoic acid (EPA) and docosahexaenoic acid (DHA) [12,13]. These omega-3 fatty acids (n3-PUFA) benefit cellular function [14,15,16], decrease inflammatory response [17,18], modulate mitochondrial biogenesis, and improve mitochondrial dysfunction [15,19]. Walnuts contain the highest amount of ALA of all nuts [20]. Other fatty acids such as oleic acid, linoleic acid, or palmitic acid provide a natural ALA-rich n3-PUFA to n6-PUFA ratio of 1:4 [21,22]. Furthermore, this extract also includes low levels of the lipophilic vitamin E, a powerful antioxidant [23,24]. Vitamin E represents an effective scavenger for reactive oxygen species (ROS) generated within the oxidative phosphorylation system of the mitochondria [3,25,26].

In the present study, we aimed to expand on our in vivo data [8] and investigated whether a lipid extract of walnuts (WE) had an effect on mitochondrial function and neuronal development in an established cellular model of early AD [10,27,28].

## 2. Materials and Methods

### 2.1. Chemicals

Chemicals used for the experiments were acquired from Merck (Darmstadt, Germany) in the highest purity available. Aqueous solutions were prepared using type-1 ultrapure water.

### 2.2. Cell Culture

Neuroblastoma SH-SY5Y (SY5Y) cells were transfected with the human APP695 coding region and used for all experiments, as previously published [10,27,28]. These SY5Y-APP695 cells were maintained in 250 mL cell culture flasks with Dulbecco’s modified Eagle’s medium (DMEM) (Gibco, Thermo Scientific, Waltham, MA, USA). The medium was supplemented with 10% (*v*/*v*) fetal bovine serum (FBS), pyruvate, non-essential amino acids, 1% minimum essential media-vitamins, penicillin, and streptomycin. Furthermore, 3 µg/mL of the antibiotic hygromycin B were added to the medium. Twice a week, cells were transferred to new culture flasks to maintain cell health and to prevent overgrowth. 

For the preparation of experiments, SY5Y-APP695 cells were collected from the flasks, counted using a Neubauer Chamber, and adjusted to 10^6^ cells/mL. Cells were then transferred into 6-well plates (qPCR, 5 × 10^5^), 24-well plates (MMP, 2 × 10^5^), and 96-well plates (ATP, ROS, and peroxidase assays, 10^5^ cells/well). After 48 h in reduced DMEM (2% FBS and other supplements identical to cultivating medium), cells were exposed to 10 µg/mL walnut extract in 12% BSA solution (WE). To assess the effect of WE on a complex-I-restricted respiratory system, cells were incubated with 25 µM rotenone 1 h after WE exposure. All cells were used for experimentation after a total of 24 h incubation.

To circumvent the low solubility of WE and to avoid the formation of lipid droplets in the medium, the extract was taken up in EtOH at a concentration 50 mg/mL and suspended in a 12% bovine serum albumin (BSA)-supplemented medium at a ratio of 2:1 [29]. Conjugation was achieved via constant rotation of the sample for 24 h at 37 °C. A fresh WE sample was regularly prepared and used for each set of experiments. Analogous to this, the same BSA concentration was also used in the control group. A set of preliminary experiments showed that 12% BSA did not affect the investigated parameters compared to a medium control. 

### 2.3. Preparation of Lipophilic Walnut Extract

Frozen walnuts harvested in California during the 2017 season were ground in an LN2-filled mortar. The samples were broken down following a protocol for Weibull–Stoldt’s acid hydrolysis. For this, 5 g of walnut powder was suspended in 150 mL 4 N HCl and boiled and stirred for 30 min. Then, 100 mL of boiling distilled water was added. The hot suspension was immediately filtered and the filter was washed with boiling water until pH-neutral. Filters and residues were dried in a drying cabinet at 105 °C for 3 h. 

Soxhlet extraction (Gerhardt Analytical Systems, Königswinter, Germany) was used for fatty acid extraction [30]. Extraction pods were pre-dried overnight and weighed. Dried filters from the previous step were placed in extraction thimbles and topped off with glass wool. Thimbles were placed in corresponding holders and mounted inside the extraction pods. Extraction pods were filled with 160 mL light petroleum before the extraction process was started. Afterward, thimbles and holders were removed. All fats were collected in the extraction pods. The pods were dried and weighed in multiple steps until the weight was constant.

Fatty acid extract was collected in glass vials and stored for further experimentation at −20 °C. 

### 2.4. Determination of Lipid and Vitamin E Composition

Fatty acid samples needed to be esterified before analysis. For this, 4 mL of saturated NaOH in MeOH was added to 100 µL of lipid extract. The mixture was heated to 80 °C for 10 min. After cooling down to r.t., 3.5 mL BF_3_ (20% in MeOH) was added to the mixture. Samples were vigorously shaken for 2 min and heated to 80 °C for 5 min. After cooling to r.t., 5 mL isooctane was added and heated to 80 °C again for 1 min. A volume of 5 mL saturated NaCl solution was added once the solution reached room temperature again. The organic phases were collected and dried of water using Na_2_SO_4_. Samples were diluted at 1:10 in isooctane and then injected into the GC/MS. Vitamin E congeners were quantified by HPLC-FD as previously published [31].

### 2.5. Cellular ROS

Cellular reactive oxygen species (ROS) levels were determined via DCFDA/H2DCFDA reaction. The cellular ROS assay kit was acquired from Abcam (Berlin, Germany) and used following the manufacturer’s guidelines. SY5Y-APP695 cells were treated with 10 µg/mL WE or control (ctrl) for 24 h at 37 °C and 5% CO_2_. Fluorescence measurments were performed using a ClarioStar plate reader (BMG Labtech, Ortenberg, Germany) at a chosen excitation/emission wavelength of 485/535 nm.

### 2.6. Peroxidase Activity Assay

Measurement of peroxidase activity in SY5Y cells was achieved using the Amplex^TM^ Red Peroxidase Kit (Thermo Fisher Scientific, Waltham, MA, USA) according to its protocols. Experiments were performed on cells sown into 96-well plates that were incubated with 10 µg/mL WE or ctrl for 24 h.

### 2.7. Quantitative Real-Time PCR (qRT-PCR) 

Cells were prepared as mentioned in Section 2.2. and washed off the well plate using PBS. Cells were pelleted at 300 g for 5 min, then resuspended in 1 mL RNAlater (Qiagen, Hilden, Germany), frozen in LN2, and stored at −80 °C until use. A RNeasy Mini Kit (Qiagen, Hilden, Germany) was used to isolate the RNA samples. 

The quality of RNA was analyzed using a NanoDrop 2000x spectrophotometer (Thermo-Fisher Scientific, Waltham, MA, USA). To enhance its quality, RNA was purified using a TurboDNA Free Kit (Qiagen, Hilden, Germany). After this, 1 µg RNA and the iScript cDNA synthesis kit (BioRad, Munich, Germany) were used for the synthesis of cDNA. Quantitative real-time PCR was performed using SYBR Green technology on a CFX96 Touch real-time PCR detection system (BioRad, Munich, Germany). Samples were analyzed in triplicate at 10 µL. Annealing temperatures, product sizes, concentrations, and sequences of primers are displayed in Table 1. Data were evaluated according to the 2(−∆∆Cq) method in BioRad CFX manager (BioRad, Munich, Germany) and adjusted to beta-actin (ACTβ), phosphoglycerate kinase 1 (PGK1), and glyceraldehyde 3-phosphate dehydrogenase (GAPDH) expression levels as recommended by the MIQE guidelines [32]. All primers were purchased from Biomol (Hamburg, Germany) or Sigma-Aldrich (Munich, Germany).

### 2.8. ATP Measurements

The ATPlite Luminescence Assay System (Perkin Elmer, Rodgau-Jügesheim, Germany) was used to measure cellular ATP levels. After cells were incubated with WE for 24 h, microwell plates were cooled to room temperature for 10 min. Following the manufacturer’s instructions, ATP concentrations were measured as emitted light in a ClarioStar plate reader (BMG Labtech, Ortenberg, Germany). The emission of light is linearly correlated to the ATP concentration and was calculated in reference to a standard curve of samples with known concentrations.

### 2.9. Mitochondrial Membrane Integrity (MMI) in SY5Y Cells

After the incubation with WE or ctrl (12% BSA solvent control), the culture medium was removed. Cells were then washed with 10 mL PBS. PBS was then replaced with 10 mL culture medium and cells were rinsed off the flask surface and triturated to break up cellular lumps. The cell suspension was pelleted at 300 g for 5 min. The supernatant was removed and cells were resuspended in 1 mL Ca-HBSS (containing 12.6 mM Ca^2+^). Cells were counted as previously described and diluted to 10^6^ cells/mL. Four reaction tubes were filled with 1 mL of WE-treated cell suspension. To each reaction tube, 10 µL Calcein AM (1 µM) and 20 µL CoCl_2_ (400 µM) were added. Finally, to one reaction tube containing control cells, 10 µL Cyclosporin A (1 µM) was added, while to a second tube, 10 µL ionomycin (50 nM) was added. The same procedure was applied for control cells in parallel. Differences in volume were balanced using the buffer. All reaction tubes were incubated for 15 min at 37 °C. Afterward, cells were centrifuged at 4 °C and 500 g for 6 min. Reactions tubes were placed on ice and the supernatant was removed. The resulting cell pellet was resuspended in 400 µL Ca-HBSS and pipetted in duplicates of 200 µL into a black 96-well plate. Fluorescence of Calcein AM was quenched by Co^2+^ following release from mitochondria into the cellular matrix via mPTP opening. Cyclosporin A was added as the negative control, inhibiting the formation of mPTP via binding to Cyclophilin D, thereby maintaining the highest fluorescence signal. Ionomycin was added as the positive control, leading to increased uptake of Ca^2+^, swelling of and rupture of mitochondria, and quenching most of the fluorescence. 

Fluorescence was measured with a ClarioStar plate reader (BMG Labtech, Ortenberg, Germany) at an excitation wavelength of 486 nm and an emission wavelength of 520 nm. Results were adjusted to protein content determined via the BCA method. 

### 2.10. Citrate Synthase Activity

Cells treated with 10 µg/mL WE or control for 24 h were collected from cell culture flasks, washed with PBS, and diluted to 10^6^ cells/mL. Samples were frozen in LN2 and stored at −80 °C until use. To measure the citrate synthase activity, samples were thawed while a reaction medium (0.1 mM 5,5′-dithio-bis-(2-nitrobenzoic acid) (DTNB), 50 µM EDTA, 0.31 mM acetyl coenzyme A, 5 mM triethanolamine hydrochloride, and 0.1 M Tris-HCl) was mixed. Then, 40 µL samples were placed into a 96-well plate. To each well, 110 µL of reaction medium was added. The microplate was heated to 30 °C for 5 min. To start the reaction, 50 µL of 0.5 mM oxaloacetate at 30 °C was to each well. Absorbance was measured in a ClarioStar plate reader (BMG Labtech, Ortenberg, Germany) at a wavelength of 412 nm for 5 min.

### 2.11. Mitochondrial Membrane Potential (MMP)

We used the fluorescence dye rhodamine-123 (R123) to investigate MMP. Following incubation with WE or control for 24 h, cells were treated with 0.4 µM R123 and kept at 37 °C and 5% CO_2_ for 15 min. Hank’s balanced salt solution (HBSS) buffer (supplemented with Mg^2+^, Ca^2+^, and HEPES; pH 7.4; 37 °C) was then used to rinse the cells and to remove excess fluorescence dye. Cells were then centrifuged at 750× *g* for 5 min. After centrifugation, the medium was aspirated and cells were resuspended in fresh HBSS. Following this, the fluorescence was measured at an excitation wavelength of 490 nm and an emission wavelength of 535 nm on a ClarioStar plate reader (BMG Labtech, Ortenberg, Germany).

### 2.12. Aβ_1–40_ Concentration

Aβ_1–40_ levels were measured using an Amyloid beta1-40 Kit (Cisbio, Perkin-Elmer, Waltham, MA, USA). Cells were treated with 10 µg/mL WE or ctrl for 24 h and collected from the flasks. PBS was used to wash cells once before they were stored in PBS containing cOmpleteTM EDTA-Free Protease Inhibitor Cocktail (Sigma-Aldrich, Munich, Germany) at −80 °C until the time of the experiment. Samples were thawed and lysed using Cell Extraction Buffer (Invitrogen, Waltham, MA, USA) before applying the Amyloid beta1–40 Kit’s protocol. A ClarioStar plate reader with HTRF filters (BMG Labtech, Ortenberg, Germany) was used to measure fluorescence. For this, emission wavelengths of 665 nm for the acceptor and 620 nm for the donor were selected. Each sample was measured in triplicate.

### 2.13. Protein Content

To determine the protein contents of previously frozen cells from MMI, CS, or Aβ_1–40_ experiments, a Pierce BCA Protein Assay Kit (Thermo Fisher Scientific, Waltham, MA, USA) was used as intended by the manufacturer. Absorbance levels of the samples were measured in a ClarioStar plate reader (BMG Labtech, Ortenberg, Germany).

### 2.14. Neurite Outgrowth in SY5Y Cells

Cells were collected from the cell culture flask, counted, and diluted to 10^5^ cells/mL. Cells were then seeded in transparent 6-well plates containing 1 mL DMEM and a microscopy coverslip. After 24 h, the medium was exchanged for reduced DMEM (2% FBS and other supplements identical to cultivating medium). Furthermore, 10 µM retinoic acid was added to start the differentiation of cells. After 5 days, the medium was changed to unsupplemented DMEM for the remaining cultivation. Additionally, cells were treated with 10 µg/mL WE or control. 

After a total of 10 days, the medium was removed and cells were washed with 2 mL PBS (1×). Cells were treated with 1 mL ROTI-Histofix ECO plus (Carl Roth, Germany, Karlsruhe) for 20 min, before being washed with 1 mL H_2_O. After this, cells were stained in 1 mL of acidified hemotoxylin solution for 1 min. Coverslips were removed from the well plate and rinsed with tap water for 15 min. Coverslips were then dipped into eosin solution for 10 s, before being passed through several washing steps in the following order: H_2_O, 70% EtOH, 90% EtOH, 100% EtOH, 100% isopropanol, and 100% xylol. Finally, one drop of highly viscous Permount (Carl Roth, Karlsruhe, Germany) was placed on a microscopy slide, then the coverslip, with cells facing down, was placed on top to completely embed cells within the mounting solution. After preparation, slides were dried overnight at room temperature before analysis via microscopy. 

Pictures were taken from moderately full areas of the sample to include at least 10 cells. From these cells, the number and length of the longest neurite were determined using Mark Longair’s Simple Neurite Tracer 3.1.4 plugin for ImageJ by an uninvolved scientist. If neurites linked two cells together, the complete neurite length was measured. 

### 2.15. Statistics

Data are presented as arithmetic means with the standard deviation (SD) or standard error of the mean (SEM). Statistical testing was performed using either Student’s t-test or a one-way ANOVA followed by a Tukey post hoc test. To calculate statistical tests, GraphPad Prism (GraphPad Software) version 8.2 for Windows was used. Outliers were removed using a ROUT outlier test by applying Q = 1.

## 3. Results

### 3.1. Characterization of Walnut Oil

Linoleic acid, oleic acid, and α-linolenic acid were the predominant fatty acids and γ- and δ-tocopherol the major vitamin E compounds in the investigated walnut lipid extract (WE) (Table 2). In total, 100 g of walnuts consisted of 63.14 ± 0.12 g of fats. 

### 3.2. Oxidative Stress Parameters

**Figure 1:**
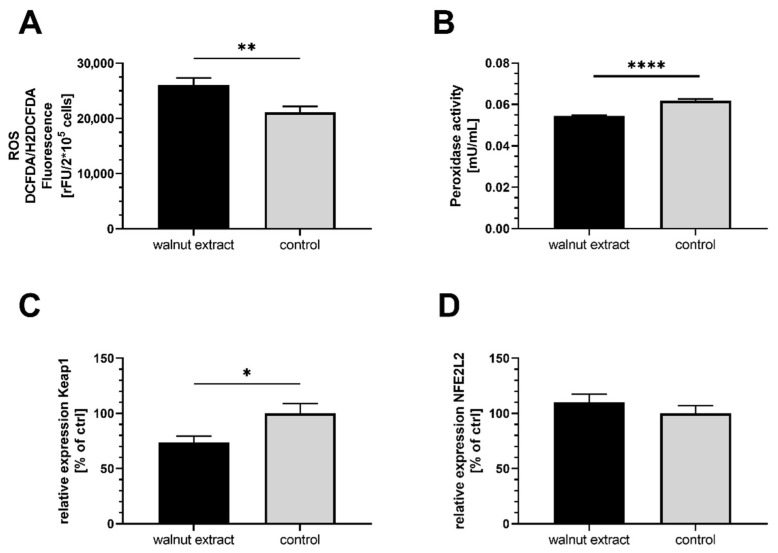
Effects of 10 µg/mL walnut fatty acid extract in SY5Y-APP695 cells. EtOH-12%BSA mixture served as control. (**A**) Cellular concentrations of reactive oxygen species (ROS) in SY5Y-APP695 cells; *n* = 8. (**B**) Peroxidase activity in SY5Y-APP695 cells; *n* = 8. (**C**) Relative mRNA expression of Keap1; *n* = 10. (**D**) Relative mRNA expression of NFE2L2; *n* = 10. PGK1, GAPDH, and ACTβ were used as reference genes according to the MIQE guidelines [32]. Data are displayed as mean ± SEM. Significant differences (student’s *t*-test) are indicated as follows: * *p* < 0.05, ** *p* < 0.01, **** *p* < 0.0001.

The cellular generation of ROS was significantly higher in SY5Y-APP695 cells treated with 10 µg/mL WE compared to ctrl (Figure 1A). ROS production in SY5Y-APP695 cells incubated with higher concentrations of WE (50 µg/mL and 100 µg/mL) did not differ from control. WE significantly decreased the peroxidase activity (Figure 1B), indicating diminished scavenging properties of the cells, which might explain the increase in ROS levels. Furthermore, relative mRNA expression of Keap1 (Figure 1C), an adaptor protein that suppresses the NFE2L2 signalling pathway and the expression of antioxidative enzymes, was reduced by WE treatment. However, expression of the downstream NFE2L2 gene was unaffected (Figure 1D). 

### 3.3. Membrane Integrity

**Figure 2:**
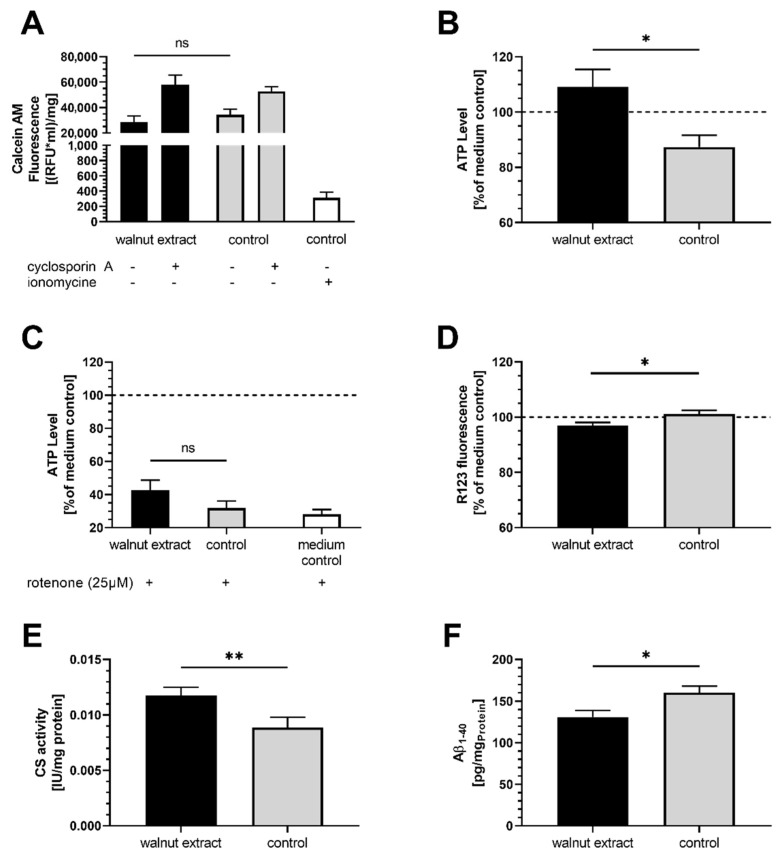
Effects of 10 µg/mL walnut lipid extract in SY5Y-APP695 cells. EtOH-12%BSA mixture served as control. (**A**) Mitochondrial membrane integrity reflected as the capacity to retain Calcein-AM (CAM) fluorescence dye. Displayed CAM fluorescence is adjusted to protein levels of the samples and reflects CAM captured in mitochondria only. Cytosolic CAM is quenched by CoCl_2_. Positive control ionomycin led to permeabilization of mitochondrial membrane and quenching of the majority of CAM. Cyclosporin A reduced the probability of opening of the mitochondrial permeability transition pore resulting in less CAM to be channeled from mitochondria to cytosol; *n* = 11. (**B**) Basal ATP concentrations; *n* = 8. (**C**) ATP concentrations following rotenone-induced (25 µM) complex I inhibition. White column represents medium control treated with rotenone; *n* = 10. (**D**) MMP determined as fluorescence of dye R123 taken up by the mitochondria; *n* = 7. (**E**) Citrate synthase activity adjusted to the protein content of the sample; *n* = 10. (**F**) Content of Aβ_1–40_ in SY5Y-APP695 cells were determined via the HTRF method and adjusted to protein levels of the samples; *n* = 9. Displayed are means ± SEM. Significant differences (student’s *t*-test) are displayed as: ^ns^ *p* > 0.05, * *p* < 0.05, ** *p* < 0.01.

Increased production of ROS could potentially lead to increased lipid peroxidation and damage to the membranes. However, the mitochondrial membrane integrity of walnut extract-treated cells was similar to control (Figure 2A). 

### 3.4. Mitochondrial Related Parameters

**Figure 3:**
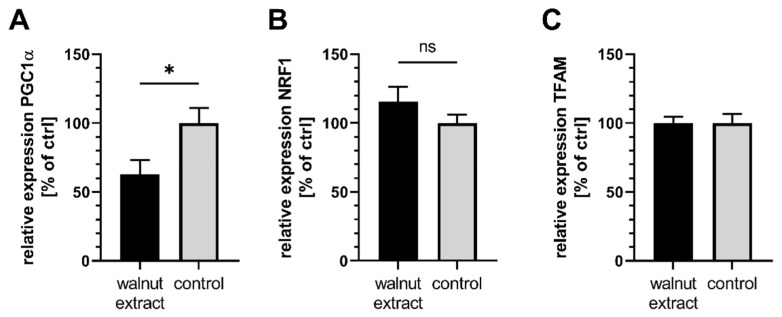
Effects of 10 µg/mL walnut lipid extract on gene expression of markers of mitochondrial biogenesis. EtOH-12%BSA mixture served as control. (**A**) Gene expression of PGC1α; *n* = 10. (**B**) Gene expression of NRF1; *n* = 10. (**C**) Gene expression of TFAM; *n* = 10. To evaluate statistical significance, a student’s *t*-test was performed in (**A**,**B**), while a Mann-Whitney test was performed in (**B**) due to data not fulfilling normality according to a Shapiro–Wilks test. Significant differences are displayed as: ^ns^ *p* ≥ 0.05, * *p* < 0.05. Results displayed as means ± SEM.

SY5Y-APP695 cells treated with WE produced significantly more ATP compared to control cells (Figure 2B). When complex I of the ETC was inhibited by the addition of 25 µM rotenone, which is commonly used in models of neurodegenerative diseases [33,34], ATP concentrations did not differ between WE-treated cells and control, but were numerically higher (Figure 2C). Mitochondrial membrane potential of cells (Figure 2D) is lower in WE-treated cells, suggesting a small amount of depolarization. Since citrate synthase activity, as a marker for mitochondrial mass [35,36], was significantly increased after WE treatment (Figure 2E), enhanced ATP levels might stem from increased mitochondrial biogenesis, which could also explain the elevated ROS concentrations (Figure 1A). In addition, increased ROS production might also stem from the reduced expression of Keap1 and its effects on antioxidation (Figure 1C). To follow up on this theory, we investigated the gene expression levels of several markers (Figure 3A–C). Expression of PGC1α was significantly lower in WE-treated cells, while NRF1 showed a trend of being increased and TFAM was virtually identical to control. Since the PGC1α/NRF1/TFAM pathway is commonly linked to mitochondrial biogenesis, these results suggest that this biogenesis is not affected. 

### 3.5. Aβ-Levels

Two hallmarks of AD are the cleavage of the amyloid precursor protein and the release of neurotoxic Aβ peptides. Treatment of SY5Y-APP695 cells with WE leads to a significant decrease in Aβ_1–40_ levels (Figure 2F). 

### 3.6. Neurite Outgrowth

**Figure 4:**
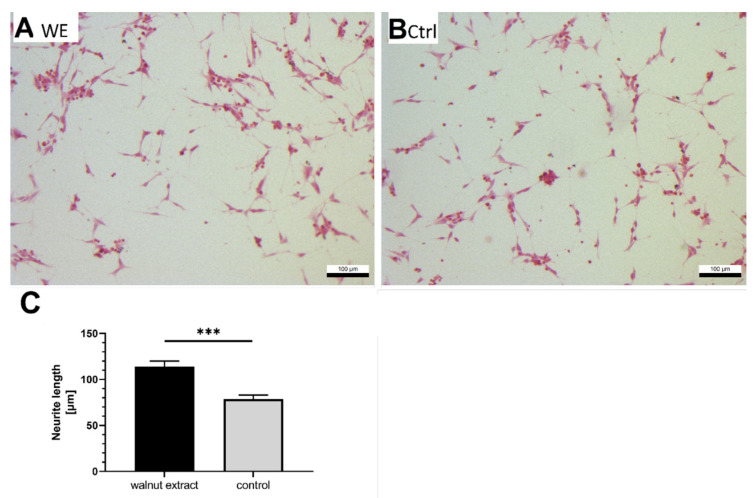
Effects of 10 µg/mL walnut extract (WE) in SY5Y-APP695 cells on neurite growth. EtOH-12%BSA mixture served as control. (**A**) Microscopic view of SY5Y-APP695 cells differentiated with retinoic acid (10 µM) and 10 µg/mL WE. (**B**) Microscopic view of cells treated with retinoic acid (10 µM) and EtOH-BSA control (Ctrl). (**C**) Quantification of neurite length of differentiated SY5Y-APP695 cells treated with 10 µg/mL WE. Data displayed as means ± SEM; *n* = 6. For each n, at least 10 neurites were measured in 3 separate images. Significance (student’s *t*-test) is displayed as *** *p* < 0.001.

In a previous study, we found that a 6%-walnut-enriched diet could improve the working memory of aged NMRI mice [8]. For this reason, we were interested in whether WE influenced the growth of neurites. We initiated the differentiation of SY5Y-APP695 cells with retinoic acid and treated cells with 10 µg/mL WE to investigate the effects on the continued growth of neurites. Treatment with WE led to significantly longer neurites in SY5Y-APP695 cells (Figure 4A–C). 

## 4. Discussion

Human neuroblastoma SH-SY5Y-APP695 cells, serving as a model for early-onset AD, were treated with a lipid extract from walnuts (WE). Analytically, we focused on fatty acids and vitamin E (VE), which have been reported to be major components of the lipid fraction of walnuts [30]. 

### 4.1. Effects on Oxidative Stress

WE increased ROS and lowered peroxidase activity in SY5Y-APP695 cells, which could have been expected since n3-PUFA are prone targets for lipid peroxidation. Schönfeld et al. reported that free PUFA increased the release of ROS in states of both normoxia and hypoxia in neuronal PC12 cells [37]. Similarly, ROS-catalyzed oxidation of dirhodamine 123 to rhodamine 123 was nearly doubled in microvascular endothelial cells upon treatment with 15 µM of different PUFA, such as EPA, DHA, or AA [38]. In yeast, treatment with 1 mM ALA led to decreased mitochondrial bioenergetics and viability, as well as an increased peroxidability index and increased ROS [39]. 

Keap1 gene expression, which is linked to the Keap1/NFE2L2/ARE pathway for antioxidative capacity, suggests a diminished ability to respond to oxidative stress. Naturally, this plays a role in the increased level of ROS but was generally not expected, as PUFA are reported to induce NFE2L2 and Keap1 [40]. Since the antioxidant response element (ARE) is only a loose term describing a plethora of different genetic sequences, its gene expression is difficult to determine [41]. For this reason, expression data have to be carefully examined. Nevertheless, lower peroxidase activity could be related to lower induction of Keap1 gene expression. Elevated ROS levels are not necessarily an indication of harmful effects. In recent years, low levels of ROS have been described to be important signaling molecules, especially in the mitochondrial electron transport chain [42,43] and as a potential inducer of hormesis [44,45]. 

### 4.2. Effects on Membranes

We assessed the quality of mitochondrial membranes and found that both WE- and control-treated cells showed the same calcein fluorescence signal, indicating that WE did not affect membrane leakage or induce damage to the membranes. Therefore, it seems unlikely that the observed increase in ROS had an effect on the membranes. Schönfeld et al. attributed the increased ROS levels found in PC12 cells to improved intracellular signalling [37]. Rather positive effects of PUFA-catalyzed lipid peroxidation were also described in a human study [46]. 

### 4.3. Role of Vitamin E

Due to the VE content, only a small increase in ROS was expected, because VE is a strong ROS scavenger [23,47] that can inhibit lipid peroxidation [48,49]. Overall, α-tocopherol represents the major congener of vitamin E and has been widely studied [48,50]. Although all congeners exhibit antioxidative properties, α-tocopherol is mainly retained in plasma [47]. In the studied WE, γ-tocopherol was the major vitamin E compound. In combination with oleic acid, γ-tocopherol reduced oxidative damage caused by 7-ketocholesterol, suggesting a protective effect on membranes [51]. Moderate concentrations of γ-tocopherol, ranging from 5 µM to 80 µM, also exceeded the effect of α-tocopherol in reducing Aβ in SY5Y cells [52]. Additionally, lower Aβ levels were associated with high γ-tocopherol concentrations determined post-mortem in the brains of AD patients [53]. The WE contained moderate amounts of 136 mg/kg γ-tocopherol and around 160 mg/kg VE in total. Taking into account the requirements for LPO protection of vitamin E content in relation to unsaturated fatty acids [54] and the presence of other antioxidants [55,56], WE should contain enough antioxidants to protect against oxidation. It is important to note that these VE requirements [54] apply to RRR-α-tocopherol and not γ-tocopherol, which might play a role in evaluating the efficacy of VE in WE. Several studies have investigated the co-supplementation of n3-PUFA with VE in human trials and have generally reported an increased total antioxidative capacity (TAC) [57,58,59]. Occasionally, increased glutathione (GSH) concentrations [58] or reduced malondialdehyde (MDA) levels are noted [57]. Furthermore, increased levels of reactive nitrogen species (RNS), similar to ROS, were observed [58,59], but did not affect MDA levels as marker for lipid peroxidation [60]. These results are in agreement with ours, which do not suggest damaged membranes. Only a few investigations have specifically examined the combination of n3-PUFA with γ-tocopherol. Himmelfarb et al. combined the n3-PUFA DHA and γ-tocopherol at a ratio of around 2.5:1 and found no effect on F2-isoprostane levels as a marker for oxidative stress in the plasma of dialysis patients [61]. 

### 4.4. Effects on Mitochondrial Function

Basal ATP was improved in SY5Y-APP695 cells following WE treatment and still tended to be elevated after rotenone addition, which inhibits mitochondrial complex I [33,62]. One explanation could be the beneficial effect of VE on mitochondrial function, as we have previously described for rice bran extracts (RBE) [63]. However, since RBE and WE have different vitamin E profiles and amounts, further experiments are needed to confirm this theory. On the other hand, it has also been described that n3-PUFA prevent ATP loss in SH-SY5Y cells after silver nanoparticle-induced damage [64] and ROS [65]. Since SH-SY5Y cells are among the few cell lines that can synthesize DHA and EPA from ALA [12], positive effects could be linked to EPA, which attenuated MPP+-induced damage [66] and protected dopaminergic neurons against MPP+/MPTP-induced cell damage in the mitochondria of rat liver cells [67]. These reports suggest that WE may have beneficial effects if cells are directly treated. Another explanation for the increased ATP concentrations after WE incubation could be a shift to other ATP-producing mechanisms such as glycolysis. Furthermore, we found a statistically significant depolarization of the MMP in WE-treated cells, which might suggest that the increased ATP production is related to an enhanced activity of the ATP synthase (complex V). The ATP synthase is driven by the MMP, more specifically a translocation of protons from the intermembrane space to the mitochondrial matrix. A small amount of depolarization could, therefore, be observed if CV is enhanced [68], while the ETC activity remains unaffected. Furthermore, slight depolarization also inhibiting excessive production of ROS has been described as a crucial component for anti-aging [69]. In this sense, the depolarization observed here might also be a counteraction against the increased levels of ROS. Another theory could be that increased mitochondrial biogenesis is responsible for increased ATP levels. Looking at key transcription factors, however, revealed that NRF1 and TFAM, encoding important parts of the mitochondria such as some complexes of the ETC [70,71,72], were unaffected by WE treatment, while the key regulator of mitochondrial biogenesis was expressed to a significantly lower extent in SY5YAPP695. In the literature, PUFA have been reported to stimulate mitochondrial biogenesis [73] in white fat and also in rat RIN-m5F cells treated with linoleic acid [74]. Linoleic acid is of paramount importance for mitochondrial biogenesis and new membrane formation, as 60–80% of cardiolipin is composed of one of its derivatives, tetra-linoleoyl cardiolipin [75]. Since 10–20% of mitochondrial membranes consist of cardiolipin, WE would be able to provide an important resource for membrane generation. These results, however, do not suggest increased biogenesis, unless PGC1α is downregulated as consequence of its prior activation. Since gene expression does not directly relate to actual protein content and PGC1α expression is described as rather short-lived [76], this might be the case, but it is rather unlikely as there should still be a significant fold change downstream of NRF1 and TFAM. Additionally, data for citrate synthase activity as a marker for mitochondrial content must be considered with caution, as the original correlation was found in skeletal muscle cells [35,77]. Furthermore, there have been reports that have linked NFE2L2 signaling and subsequent antioxidative response to PGC1α as well, suggesting that our observed reduced peroxidase activity might originate from the lower expression of PGC1α [78,79,80]. 

In this sense, we believe it is more likely that increased ATP stems from enhanced CV activity.

### 4.5. Effects on Aβ Production

Similar to our previous work, which showed that the supplementation of the n3-PUFA DHA increased sAPPα and reduced Aβ production [81], we investigated whether WE would affect Aβ concentrations. Treatment of cells with 10 µg/mL WE resulted in significantly lower Aβ_1–40_ concentrations. PUFAs such as DHA, EPA, ALA, LA, OA, and ARA inhibit the aggregation of Aβ_1–40_ and Aβ_1–42_ by up to 84% [82], while DHA, in combination with tocopherol, lowered Aβ_1–42_ in APP-transfected CHO cells [83]. This is also in line with findings that DHA lowered the Aβ burden in a model of aged APPsw transgenic mice [84]. 

### 4.6. Effects on Neuritogenesis

We recently demonstrated that feeding a 6%-walnut-enriched diet to aged NMRI mice led to significantly improved spatial memory [8]. However, we were unable to link this observation directly to mitochondrial function or altered gene expression for neuronal function [8]. Based on these findings, we examined the effect of WE on neurite growth after retinoic acid differentiation in SH-SY5Y-APP695 cells. Indeed, neurites were significantly longer after WE treatment. Although amyloidogenic cleavage of APP is well described and documented as a hallmark of AD, the physiological function of APP protein is still a matter of debate. However, several reviews have generally linked APP to neurite growth and development [85,86,87]. For this reason, SY5Y-APP695 cells would be expected to grow longer neurites compared to non-transfected cells, which is indeed the case considering the average neurite length of 30–60 µm [88,89]. Since a transfection with APP695 not only introduces APP into the cells but also leads to increased production of Aβ_1–40_ and Aβ_1–42_, these entities have to be considered as well. Contrary to APP, amyloid-beta peptides have been generally considered to counteract neurite growth [90,91]. Petratos et al. specifically found this to be the case in SH-SY5Y cells [91]. The increased neurite length is, therefore, in good agreement with our results and indirectly supports the reduced Aβ_1–40_ levels we found. Because of this reduction, neurite growth may be less affected by Aβ and may result in much longer neurites compared with non-transfected SH-SY5Y cells. Neurite growth plays an important role in neuronal function and development, which also suggests a beneficial effect of WE. 

### 4.7. Limitations

There are limitations to our study. The lipid extract used in the current study could potentially contain minor amounts of other lipophilic compounds as well. Examples include low amounts of squalene and phytosterols, such as campesterol, stigmasterol, and β-sitosterol [56]. While phytosterols are known to lower LDL-cholesterol [92], squalene was reported to improve mitochondrial function and induce peroxidase activity in the livers of rats [55]. Because we observed reduced peroxidase activity after WE treatment in neuroblastoma cells, and since our extraction method resulted in the enrichment of n3-PUFA and VE (Table 1), we assume that the effects reported herein are mainly due to n3-PUFA and VE as major components of WE. Another limitation is the fact that this cellular model was used, as this was planned to be a follow-up investigation of our study in animals [8]. Similar to mice characterized by a higher conversion rate of ALA to EPA and DHA compared to humans [93,94,95], SH-SY5Y cells are also able to facilitate this conversion [12,13]. In the same sense, as it is difficult to translate effects of animal diets to humans [95], the observed effects here should also be understood as preliminary. Finally, most of WE was made up of n3-PUFA, and as mentioned before these have shown beneficial effects on aging and neuronal function. However, next to their original properties, n3-PUFAs can also be metabolized to a vast array of bioactive components, namely so-called oxylipins [96,97], of which only a few are fully characterized. This opens up a broad spectrum of functions and potential targets that need to be investigated in the future. 

## 5. Conclusions

WE might increase ATP production due to enhanced complex V activation, while CI-IV remains unaffected. Decreased Aβ_1–40_ formation and enhanced ATP levels, which could be linked to enhanced neurite growth, suggest that a lipophilic walnut extract has beneficial properties on neuronal development and AD-associated amyloid-beta in a cellular model of early AD. Further research is necessary to link the observed effects to specific ingredients of the extract and to what extent these results can be translated to the human populace. 

## Figures and Tables

**Table 1:** Primer sequences, size of products, concentrations, and the protocols used for qRT-PCR measurement. Housekeeping genes were ACTβ, PGK1, and GAPDH.

Primer	Sequence	Size [bp]	Conc [µM]	Annealing Temp. (Time) (Cycle No.)
ACTβ	5′-GGACTTCGAGCAAGAGATGG-3′ 5′-AGCACTGTGTTGGCGTACAG-3′	234	0.2	58 °C (30 s), (45×)
PGK1	5′-CTGTGGGGGTATTTGAATGG-3′ 5′-CTTCCAGGAGCTCCAAACTG-3′	198	0.2	58 °C (30 s), (45×)
GAPDH	5′-GAGTCAACGGATTTGGTCGT-3′ 5′-TTGATTTTGGAGGGATCTCG-3′	238	0.2	58 °C (30 s), (45×)
Keap1	5′-GCACAACTGTATCTATGCTG-3′ 5′-CTCCAAGGACGTAGATTCTC-3′	167	0.45	58 °C (30 s), (45×)
NFE2L2	5′-CGTTTGTAGATGACAATGAGG-3′ 5′-AGAAGTTTCAGGTGACTGAG-3′	122	0.3	58 °C (30 s), (45×)
PGC1α	5′-CATCCCTCTGTCATCCTC-3′ 5′-GCAGACCTAGATTCAAACTC-3′	146	0.2	60 °C (30 s), (45×)
NRF1	5′-GTAACCCTGATGGCACTGTC-3′ 5′-TCTGGATGGTCATCTCACT-3′	183	0.2	58 °C (45 s), (45×)
TFAM	5′-TCCCCCTTCAGTTTTGTGTA-3′ 5′-ATCAGGAAGTTCCCTCCAAC-3′	189	0.4	58 °C (30 s), (45×)

**Table 2:** Detailed fatty acid and vitamin E congener composition of the walnut extract. Fat content was determined gas-chromatographically. The cut-off for signals was at 0.05% of the strongest signal obtained. Data are displayed as g/100 g extract for fatty acids and mg/100 g for vitamin E congeners. Tocopherols were determined by HPLC/FD. Note: n.d. = not detected; SFA = saturated fatty acids; MUFA = mono-unsaturated fatty acids; PUFA = poly-unsaturated fatty acids.

Fatty Acids		Concentration ± SD [g/100 gWE]
Palmitic acid	16:0	7.30 ± 0.042
Stearic acid	18:0	2.59 ± 0.085
Oleic acid	18:1	17.1 ± 0.078
Vaccenic acid	18:1 *trans*	0.81 ± 0.014
Linoleic acid	18:2	57.8 ± 0.014
α-Linolenic acid	18:3	14.5 ± 0.021
SFA		9.89 ± 0.042
MUFA		0.81 ± 0.014
PUFA		89.3 ± 0.071
**Vitamin E**		**Concentration ± SD [mg/100 gWE]**
α-tocopherol		n.d.
β-tocopherol		n.d.
δ-tocopherol		2.24 ± 0.20
γ-tocopherol		13.6 ± 1.02
α-tocotrienol		n.d.
β-tocotrienol		0.037 ± 0.004
δ-tocotrienol		n.d.
γ-tocotrienol		0.097 ± 0.032
Total vitamin E		15.98 ± 1.25

## Data Availability

The dataset generated during this study is available from the corresponding author upon reasonable request.
